# Supplementary material for: Acanthosis nigricans as a composite marker of cardiometabolic risk and its complex association with obesity and insulin resistance in Mexican American children
Source: PLoS One. 2020 Oct 15;15(10):e0240467. doi: 10.1371/journal.pone.0240467 (PMC7561152; doi:10.1371/journal.pone.0240467)
Supplement: S1 Table — (DOCX) [file pone.0240467.s001.docx]

**Supplement Table 1. Types and numbers of relative pairs among 673 SAFARI children and adolescents, aged 6 to 17 years old**

| **Type of Relative Pair** | **No. of Pairs** |
| --- | --- |
| Siblings | 383 |
| Avuncular | 9 |
| Half-sibs | 86 |
| Half-avuncular | 5 |
| 1^st^ cousins | 550 |
| 1^st^ cousins, 1 rem | 234 |
| Half first cousins | 74 |
| 1^st^ cousins, 2 rem | 2 |
| Half first cousins, 1 rem | 36 |
| 2^nd^ cousins | 661 |
| 2^nd^ cousins, 1 rem | 512 |
| Half second cousins | 178 |
| 3rd cousins | 662 |
| Half second cousins, 1 rem | 10 |
| 2^nd^ cousins, 2 rem | 6 |
| 3rd cousins, 1 rem | 137 |
| Half third cousins | 6 |
| 4^th^ cousins | 30 |
| Other relatives (third and fourth cousins, double third cousins and 2^nd^ cousins, half-avuncular, and others) | 83 |
| Total | 3,664 |
