# Supplementary figures and images for: Acanthosis nigricans as a composite marker of cardiometabolic risk and its complex association with obesity and insulin resistance in Mexican American children
Source: PLoS One. 2020 Oct 15;15(10):e0240467. doi: 10.1371/journal.pone.0240467 (PMC7561152; doi:10.1371/journal.pone.0240467)

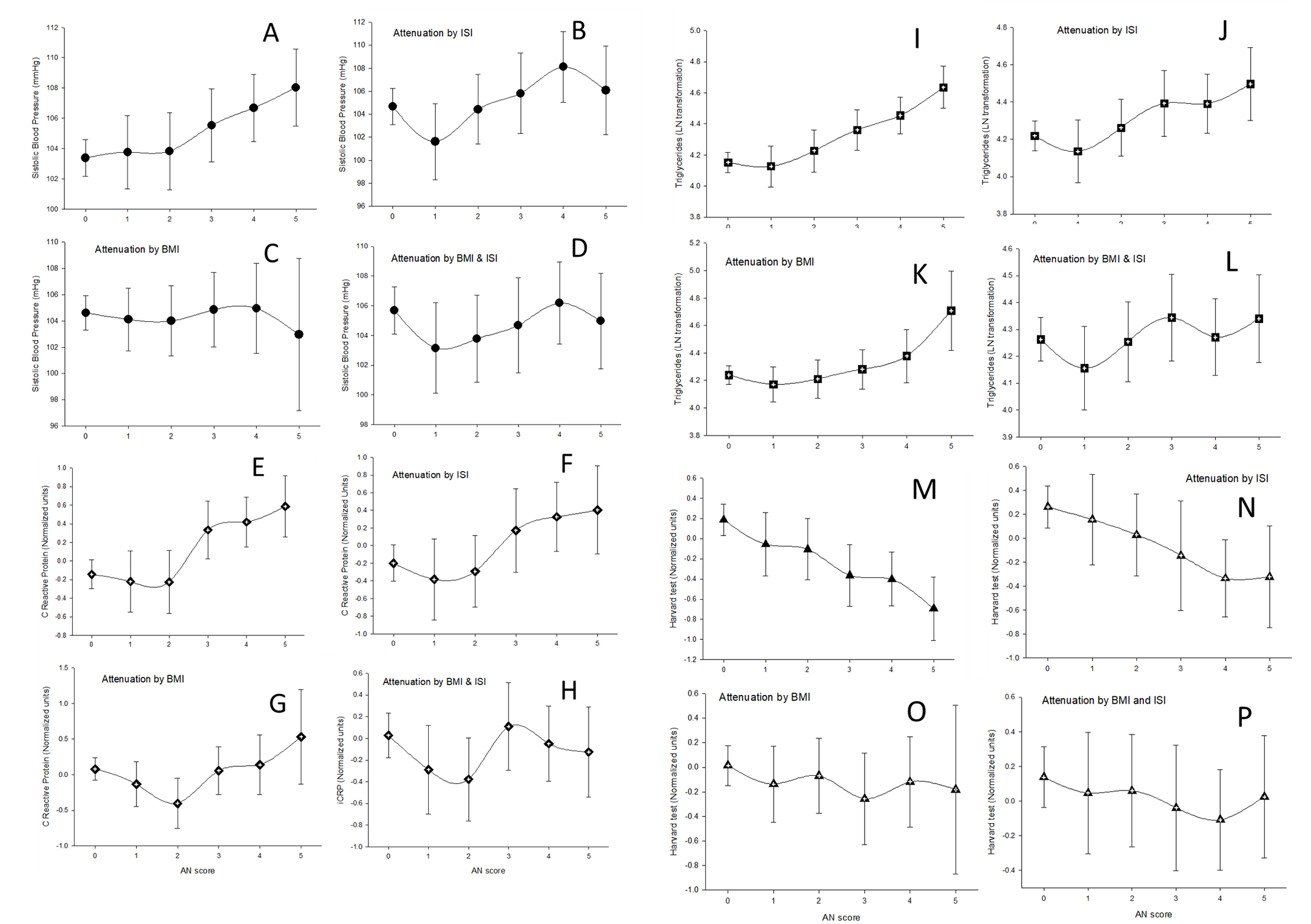

Supplement: S1 Fig — Panels A to D represent systolic blood pressure; E to H hsCRP; I to L triglycerides, and M to P PFS (Harvard test). The phenotypic function by ANc adjusted by sex, age and family is shown in the panels A, E, I and M. These functions shows clear positive or inverse relationship with ANc. The attenuation by BMI is shown in panels C, G, K and O; the hsCRP was maintained in a cubic polynomial and triglycerides on squared. The hsCRP shows a critical point between scores 2 to 3 showing a clear increase on the concentration; meanwhile triglycerides was positive for both BMI and ISI variables. When analyzed with the two covariates, all variables were attenuated. (TIF) [file pone.0240467.s002.tif]
