# Supplementary material for: Acanthosis nigricans as a composite marker of cardiometabolic risk and its complex association with obesity and insulin resistance in Mexican American children
Source: PLoS One. 2020 Oct 15;15(10):e0240467. doi: 10.1371/journal.pone.0240467 (PMC7561152; doi:10.1371/journal.pone.0240467)
Supplement: Attachment — Submitted filename: Response to Reviewers 20Aug20.pdf [file pone.0240467.s006.pdf]

The University of Texas
Rio Grande Valley
School of Medicine
South Texas Diabetes and Obesity Institute
1201 W. University Dr., EREBL Rm. #2.212
Edinburg, TX 78539
(956) 665-6478

Edinburg, Texas August 19, 2020

Fernando Guerrero-Romero, MD, PhD
Academic Editor
PLOS ONE

Reference: **PONE-D-20-02036** “Acanthosis Nigricans as a Composite Marker of Cardiometabolic Risk and Its Complex Association with Obesity and Insulin Resistance in Mexican American Children”.

Dear Dr. Guerrero-Romero,

We thank you and the reviewers for the constructive and valuable comments and suggestions. We have responded to the concerns and suggestions point-by-point as suggested. We believe that our responses to your comments and their incorporation in the revised manuscript have improved the nature of the manuscript greatly.

Following your suggestion, we are submitting the following files: :

1. Response to Reviewers
2. Revised Manuscript with Track Changes
3. Manuscript

Here below are our response to the questions related to “Journal Requirements”:

A. Journal Requirements:

1. Please ensure that your manuscript meets PLOS ONE's style requirements.

Response: We made changes to ensure that our manuscript meets PLOS ONE’s style requirements.

2. We note that you have included the phrase “data not shown” in your manuscript. Unfortunately, this does not meet our data sharing requirements.

Juan Carlos Lopez Alvarenga, MD, DSc
Assistant Professor, School of Medicine
juan.lopezalvarenga@utrgv.edu

Response: We thank you for the comment/requirement, and we have now included the relevant information in Supplemental Figure 1. Thus, the phrase “data not shown” is replaced by “Supplemental Figure 1” in the manuscript.

3. In your Data Availability statement, you have not specified where the minimal data set underlying the results described in your manuscript can be found.

Response: Following the comment, we have now provided supplementary files relating to the minimal data sets corresponding to the results including the matrices of genetic and environmental correlations and the data of residuals (i.e., residuals obtained for a given phenotype after adjusting for covariate effects) that we used for the analyses. None of them have any identification codes.

4. We note that you have provided funding information that is not currently declared in your Funding Statement. However, funding information should not appear in the Acknowledgments section or other areas of your manuscript.

Response: We are sorry for the confusion. We have now correctly provided/updated funding information in our Funding Statement and removed all funding-related information from the manuscript. We have now clarified/stated that “The funders had no role in study design, data collection and analysis, decision to publish, or preparation of the manuscript”, and provided information on the names of individuals that received salaries from specific funders. As suggested, we are including the following amended statement in this cover letter as follows:

This study was supported RD received grants: R01 HD049051/HD049051-5S1 [ARRA], HD041111, DK053889, DK042273, DK047482, MH059490, P01 HL045522, M01-RR-01346, and Veterans Administration Epidemiologic Grant. The funders had no role in study design, data collection and analysis, decision to publish, or preparation of the manuscript. Some of the investigators received salaries from some of the grants as follows: R01 HD049051/HD049051-5S1 [ARRA] (RD, DEH, JB, RAD, CPJ, RA, SP, VSF, SPF, RGR), HD041111 (RD, JB, RAD, CPJ, RA, RGR), DK053889 (RD, JB, CPJ, SP, VSF, SPF, RGR), DK042273 (DML, RD, JB), DK047482 (DML), MH059490 (JB), P01 HL045522 (JB, RD), and Veterans Administration Epidemiologic Grant (RAD, CPJ).

5. PLOS requires an ORCID iD for the corresponding author.

Response: As required, we have now provided the ORCID ID for the corresponding author Dr. Juan Carlos Lopez-Alvarenga.

B. Review Comments to the Authors:

Reviewer 1 Comment 1 (R1C1): […] The relationship among these factors is not novel, but the authors have a novel statistical approach using many variables, genetics, and phenotypic. They found that obesity explains the association of IR with AN, but no causal relationship between IR and AN in Mexican American children. The results of this study

Juan Carlos Lopez Alvarenga, MD, DSc
Assistant Professor, School of Medicine
juan.lopezalvarenga@utrgv.edu

could be important in clinical practice due to the AN severity-classification, which is low-cost, and it seems to be easy to measure, is correlated with cardiometabolic factors […].

Reviewer 1 Response 1 (R1R1): We thank the reviewer for the critical comment on our data and results in a positive way, and the potential significance of AN severity-classification in clinical practice.

R1C2: The kinship of the sample is not describing in the manuscript; I mean the pairs of full or half-brothers, cousins, etc. […] It is also not clear if hereditability is the only genetic variable used in the study.

R1R2: Following the comment, we have now provided information on various types of relative pairs in the manuscript, described in our previous publication by Fowler et al. (reference 10). In addition to the heritability estimate, as stated in our manuscript, we have used information on genetic and environmental correlations obtained from the bivariate genetic analyses, which were subsequently used for the mediation analyses (as described under 2.2 and 2.3 of Material and Methods sections of the manuscript).

R1C3: Describe criteria cut-off for prediabetes and cardiometabolic factors [dichotomized IFG, IGT, HDL-C, blood pressure and son on].

R1R3: We have now described the criteria/cut-offs used for various dichotomous traits used for the analyses.

R1C4: Which were the environmental variables used? Those need clarification.

R1R4: We clarify that the phenotypic correlation between a given trait-pair (e.g., BMI and AN-q) is partitioned into additive genetic and random environmental components using the bivariate genetic analysis, phenotypic data, and pedigree information. The additive genetic correlation is a measure of common or shared genetic basis (i.e., pleiotropy) of a trait-pair, while the random environmental correlation is a measure of the strength of the correlated response of a trait-pair to non-genetic factors.

R1C5: The authors mention three methods to choose the best model (goodness-of-fit statistics, Akaike information criterion, and Bayes information). Still, they did not specify the threshold criterion to select the models. For example, mention the difference in the AIC values. This is a concerning matter for the reproducibility and the credibility of the chosen best model.

R1R5: Following the reviewer's comment, we have now provided information related to the threshold criteria used to select parsimonious models specific to a given approach (i.e., AIC and BIC).

R1C6: Mediation analysis has enough statistical power, but what about with the variance components analysis?

Juan Carlos Lopez Alvarenga, MD, DSc
Assistant Professor, School of Medicine
juan.lopezalvarenga@utrgv.edu

R1R6: We have now reported that we will have 80% power to detect heritabilities as small as 0.25, given our sample size and pedigree structure used for this study. Regarding power to detect genetic correlations in our data, we will have 86% power for detecting a genetic correlation as small as 0.30.

R1C7: For clinical practice, are the pediatricians familiarized with the AN severity scale used in this study? If they are not, extending the AN as a proxy of cardiometabolic factors could be a limitation.

R1R7: We thank the Reviewer for the interesting comment. Pediatricians are familiar with AN, especially with that associated with insulin mutations or polycystic ovary syndrome. Recently, guidelines for treatment of obesity include the clinical inspection of AN. As described in our manuscript, the state of Texas has mandated programs across schools for AN assessment to identify children who may be at risk of developing type 2 diabetes and its related conditions.

R2C1: Please add a brief discussion on the ethnicity of the experimental group, in addition to describing them as Mexican Americans please provide more information on the ethnic ancestry of their two previous generations. It is also relevant to know if they were born in the US, if they are US citizens, or if they were born in Mexico and migrated to the US. This would provide a better understanding on the possible applications of the reported results to Mexican children living in urban areas similar to the state of Texas, such as the nearby city of Monterrey, Nuevo Leon, Mexico.

R2R1: We thank the Reviewer for the interesting comment. As stated now in the manuscript, the parental studies (SAFHS, SAFDGS, VAGES) of our SAFARI study were initiated in the early 1990s. However, based on a subset of the SAFARI children (~70%) for whom the information on birth places of mothers (and maternal grandparents) and fathers (and paternal grandparents) was available, some parents (i.e., mother or father) of the SAFARI children were born outside of the US, almost exclusively from Mexico as follows: Mothers = ~8% (grandmother = 19% and grandfather = 22%) and Fathers = ~12% (grandmother = 23% and grandfather = 26%).

6. While revising your submission, please upload your figure files to the Preflight Analysis and Conversion Engine (PACE) digital diagnostic tool, https://pacev2.apexcovantage.com/.

Response: We have followed your suggestion/requirement regarding conversion of figure files, and thank you.

Juan Carlos Lopez Alvarenga, MD, DSc
Assistant Professor, School of Medicine
juan.lopezalvarenga@utrgv.edu
